# Deprivation, timing of preschool infections and *H. pylori* seropositivity at age 49-51 years: the Newcastle thousand families birth cohort

**DOI:** 10.1186/1471-2334-13-422

**Published:** 2013-09-08

**Authors:** Mark S Pearce, David I Campbell, Kay D Mann, Louise Parker, Julian E Thomas

**Affiliations:** 1 Institute of Health & Society, Sir James Spence Institute, Newcastle University, Royal Victoria Infirmary, Newcastle upon Tyne NE1 4LP, UK; 2 Sheffield Children’s NHS Foundation Trust, Sheffield, UK; 3 Departments of Medicine and Paediatrics, Dalhousie University, Halifax, Nova Scotia, Canada

**Keywords:** Helicobacter pylori, Socio-economic status, Measles, Chicken pox, Mumps

## Abstract

**Background:**

*Helicobacter pylori* infection is acquired in early childhood and persists for life (or until eradication treatment is taken). Seropositivity of *H. pylori* at age 49-51 years was assessed in relation to socio-economic deprivation in early life and the timing of other childhood infections common at that time.

**Methods:**

Prospectively collected socio-economic and morbidity data from the Newcastle Thousand Families study, a birth cohort established in 1947. *H. pylori* IgG seropositivity was assessed at 49-51 years and examined in relation to both whether the individual had been diagnosed with one of measles, mumps or chicken pox, and, if so, the age at first infection. This was done in logistic regression models, allowing adjustment for socio-economic status and housing quality in childhood.

**Results:**

Adult *H. pylori* status was strongly linked to disadvantaged socio-economic status in early life (p ≤ 0.002), unlike measles, mumps and chicken pox which showed no associations. Early measles infection was independently associated with *H. pylori* seropositivity (p = 0.01).

**Conclusions:**

Of the four infectious diseases that we have studied, it appears that *H. pylori* differs from the others by the strength of association with socio economic deprivation in early childhood.

Our findings further highlight the complex interaction between measles, childhood infections and other non-microbiological factors that occur within a whole population. These data suggest a strong association between H. pylori and deprivation and raise the possibility of an interaction between early measles exposure and increased risk of exposure to *H. pylori* infection.

## Background

Chronic *Helicobacter pylori* infection is common amongst adults throughout the world, and is associated with low socio-economic status and overcrowded inadequate housing in childhood [1-6]. While a decline in prevalence was seen in developed countries [7-9], likely due to improvements in daily quality of living and hygiene, relatively lower levels of infection remain in developed countries [10], but high levels remain in developing countries [6].

Helicobacter colonization is almost certainly acquired from close contact with infected individuals in the early years of childhood, persists for decades, and becomes a major risk factor for the development of gastroduodenal disease in adult life [11]. The most serious of these diseases is gastric cancer, and for this reason *H. pylori* has been classified as a class I carcinogen since 1994 by the World Health Organisation [12,13]. It is unclear whether childhood socio-economic deprivation predisposes specifically to *H. pylori* infection, or leads to an overall increased risk of acquiring infections in early life or whether it is linked to the timing of other infections. Understanding the timing of infection risks in childhood in relation to *H. pylori* may lead to opportunities to intervene in early gastric carcinogenesis, an adult disease which appears to have a childhood origin. Investigating the potential interactions between common childhood viral infections and *H. pylori* seropositivity risk is important to this understanding. This is particularly the case in populations where both *H. pylori* and other childhood infections remain common.

We investigated the contribution of socio economic deprivation (scored by housing deprivation and paternal occupation (at birth and 5 years of age) and a documented history of measles, mumps and chicken pox (including timings of infections) to the risk of *H. pylori* seropositivity in the Newcastle Thousand Families Study at 49-51 years of age.

## Methods

The Newcastle Thousand Families study, details of which are reported elsewhere [14,15], provided a unique opportunity to compare prospectively collected records of early childhood morbidity including timings and socio-economic status, with adult *H. pylori* seropositivity. The birth cohort began as a prospective study of all 1142 children born in May and June 1947 to mothers resident in Newcastle upon Tyne, UK, covering the entire social spectrum of the city at that time. The health, growth and development of the cohort were followed in great detail up to age 15 years. Throughout the first years of the children’s lives, all families were visited both on a routine (up to every six weeks during infancy and at least quarterly until age five years) and on an *ad hoc* basis by the study team, which consisted of health visitors (nurses who visited families at home) and paediatricians.

Socio-economic status (I, (the most advantaged), II, III non-manual, III manual, IV and V (the least advantaged)) during childhood was derived from the 1990 UK Registrar General’s Standard Occupational Classification using paternal occupation at birth and the occupation of the main wage earner in the household at age 5 years, both recorded prospectively. Unemployed fathers were grouped within the least advantaged social class (V). Housing conditions at birth were assessed by a housing survey carried out by the Public Health Department in the City, and scored, on a scale of 0–4, for the presence of overcrowding, lack of hot water, shared toilet and dampness or poor repair (i.e. one point for each of the factors present). Regular visits were made by health workers and records made of any illness occurring in children, whilst GPs notified the study team of any consultations that occurred with study members. Recorded illnesses included infections of measles, chicken pox and mumps from birth up to the age of 15 years.

The cohort underwent a major follow-up at age 49-51 years [14,15]. Participants in this investigation were cohort members who were either traced through the National Health Service Central Register or contacted the study team in response to media publicity in the mid 1990’s. Between October 1996 and December 1998 self-completion questionnaires on health and lifestyle were sent out and study members invited to attend for clinical examination.

From the original cohort of 1142, 967 remained in the study to age one year, of whom 44 died before age 50 years. From the 923 survivors, 832 were traced. Of these, 574 returned a self-completion questionnaire, 412 attended for a health assessment, and 407 (44% of the surviving sample) had a blood sample taken for inclusion in this project. It has been previously been demonstrated that this sample are representative of the original cohort in terms of the data available in early life [16].

*H. pylori* IgG levels were measured by ELISA as previously described [17]. Briefly, crude whole cell antigen was prepared from pooled isolates of H. pylori cultured from gastric biopsies taken from five adults who had all undergone diagnostic upper endoscopy for dyspepsia. Sera samples from study members were diluted in blocking buffer (phosphate buffered saline, pH 7.4) + 0.05% (vol/vol) Tween 20 +1% (wt/vol) bovine serum albumin), and absorbed overnight at 4°C against crude Campylobacter jejuni antigen prior to assay. Diluted and absorbed sera were added to washed antigen-coated wells on microtitre plates, and the assay conducted as previously reported [17]. Control sera were obtained from individuals of known *H. pylori* status, chosen to cover the useful range of the assay. Individual plates were calibrated against the mean values for each of the six control sera, one of which had previously been shown to represent the cut-off value distinguishing positive from negative results [17]. Specific IgG levels greater than this cut-off control were therefore designated seropositive, and those below seronegative.

The study received ethical approval from the South Durham Lead Research Ethics Committee and the Joint Newcastle Health Authority / University of Newcastle upon Tyne Ethics Committee, and all study members gave their written informed consent. The study complied with the Helsinki declaration.

### Statistical analysis

Social class at birth and at ages 5 and 49-51 years was collapsed into three categories; most advantaged (I,II); middle (3 non-manual, manual); least advantaged (IV,V), due to small numbers in the extreme classes. Housing conditions and social class at all ages were treated as categorical variables. Measles, mumps and chicken pox infection in childhood were recorded as binary variables with corresponding age at initial infection treated as continuous for each infection type. Logistic regression was used to assess the associations between *H. pylori* seropositivity and housing deprivation, social economic position and childhood infection, and between housing deprivation and socio-economic position with childhood infection. Odds ratios (OR) and corresponding 95% confidence intervals (95% CI) are given. The Wilcoxon rank sum test was used to assess associations between age at each type of initial childhood infection and *H. pylori* seropositivity, restricted to those that had the infection. All statistical analyses were done in the statistical software package Stata, version 12 (Statacorp; College Station, TX).

## Results

Of the 407 individuals, 62 left the study during the first 15 years, but returned for follow-up aged 50, when 161/407 (40%) were seropositive for *H. pylori*. We therefore had incomplete childhood health records for these 62 individuals. In total, 344 were recorded as suffering from measles during childhood and only 32 children followed-up to age 15 years remained free of the disease. For those individuals for whom complete records existed, 223/357 developed chickenpox, and 130/350 mumps, before age 15 years.

Seropositivity to *H. pylori* was significantly associated with adverse childhood living conditions, and with increasing socio-economic disadvantages at birth and ages 5, and 49-51 years (Tables 1 and 2). The association between early socio-economic status and other childhood infections was less clear-cut (Table 3). There was a significant association between measles infection and social class at birth (p = 0.02, the greatest odds of measles in the ‘middle’ social classes), but not with social class at age 5 years, or housing deprivation scores at any age. No significant trends were seen between chickenpox infection and housing deprivation score or social class at age 5 years, although a significant trend was seen between chicken pox infection and social class at birth (decreasing risk with more affluent economic status, p = 0.004). No significant associations or trends were detected between mumps infection and social class or housing deprivation score at any age.

**Table 1:** **The association between adult *H. pylori *seropositivity and housing deprivation scores at birth and 5 years (scored one each for the presence of for the presence of overcrowding, lack of hot water, shared toilet, dampness or poor repair)**

**Housing deprivation score**	**At birth**		**At age 5 years**	
	**Proportion of *H. pylori *+ ve subjects**	**OR (95% CI)**	**Proportion of *H. pylori *+ ve subjects**	**OR (95% CI)**
0 (least deprived)	68/198 (34%)	Reference	67/193 (35%)	Reference
1	41/102 (40%)	1.28 (0.79,2.10)	45/103 (44%)	1.46 (0.89,2.38)
2	29/58 (50%)	1.91 (1.06,3.46)	23/40 (58%)	2.54 (1.27,5.09)
3 (most deprived)	21/47 (45%)	1.54 (0.81,2.94)	14/29 (48%)	1.76 (0.80,3.85)
p value for trend	0.145		0.036	

**Table 2:** **The association between adult *H. pylori *seropositivity and socio-economic occupation group in childhood and at age 49-51 years**

**Socio-economic occupation group**	**At birth**		**At age 5 years**		**At age 50 years**	
	**Proportion of H. pylori + ve subjects**	**OR (95% CI)**	**Proportion of H. pylori + ve subjects**	**OR (95% CI)**	**Proportion of H. pylori + ve subjects**	**OR (95% CI)**
Most advantaged	7/43 (16%)	0.33 (0.14,0.77)	9/29 (31%)	0.77 (0.33,1.78)	54/196 (28%)	0.41 (0.26,0.65)
Middle	91/246 (37%)	Reference	74/201 (37%)	Reference	65/135 (48%)	Reference
Least advantaged	59/110 (54%)	1.97 (1.25,3.11)	60/126 (48%)	1.56 (0.99,2.45)	31/52 (60%)	1.59 (0.83,3.04)
p value for trend	<0.001		0.088		<0.001	

**Table 3:** Associations of childhood infection with socio-economic and housing deprivation groups

	Socio-economic occupation group*			**Housing deprivation score†**	
	**At birth**	**At age 5 years**		**At birth**	**At age 5 years**
Measles					
1	0.44 (0.13,1.44)	0.60 (0.16,2.26)	0	Reference	Reference
2	Reference	Reference	1	1.21 (0.48,3.07)	0.54 (0.25,1.21)
3	0.31 (0.14,0.70)	0.51 (0.24,1.12)	2	0.97 (0.39,2.78)	0.71 (0.22,2.28)
			3	1.29 (0.36,4.65)	0.20 (0.28,17.5)
p-value for trend	0.015	0.235		0.955	0.304
Chicken pox					
1	2.36 (0.93,5.98)	0.99 (0.42,2.33)	0	Reference	Reference
2	Reference	Reference	1	0.75 (0.44,1.27)	0.72 (0.44,1.18)
3	0.57 (0.35,0.93)	0.64 (0.40,1.01)	2	0.71 (0.38,1.36)	0.55 (0.27,1.10)
			3	0.69 (0.35,1.38)	1.16 (0.50,2.69)
p-value for trend	0.004	0.149		0.543	0.234
Mumps					
1	1.22 (0.57,2.59)	0.68 (0.27,1.72)	0	Reference	Reference
2	Reference	Reference	1	1.17 (0.69,2.00)	1.82 (1.10,3.03)
3	0.60 (0.35,1.00)	0.74 (0.46,1.18)	2	1.07 (0.56,2.03)	1.21 (0.58,2.49)
			3	0.68 (0.33,1.43)	1.52 (0.68,3.39)
p-value for trend	0.094	0.372		0.600	0.123

*1: most advantaged, 2: middle, 3: least advantaged.

† 0: least deprived 3: most deprived.

There was a significant association between the age of first measles infection and adult *H. pylori* seropositivity (Wilcoxon p = 0.01). The median age of first measles infection was 4.0 years amongst adults seronegative for *H. pylori*, and 3.2 years amongst those who were seropositive. A logistic regression model showed a decreasing trend of risk of positive *H. pylori* status at age 49-51 years with increasing age of first measles infection (continuous OR 0.86 per year, 95% CI 0.76-0.98, unadjusted p = 0.02). The association remained significant after adjusting for social class at birth (OR 0.88 per year, 95% CI 0.77-1.00, p = 0.05). There were no significant associations between age of first measles infection and either social class or housing score at either birth or five years.

We observed a trend towards an association between childhood chickenpox and adult *H. pylori* seropositivity that did not achieve statistical significance (p = 0.07), but there was no association between the age of chickenpox infection and adult *H. pylori* status. The median age of chickenpox infection amongst the *H. pylori* positive group was 5.5 years, and 5.2 years amongst the negatives. There was also no significant association between *H. pylori* seropositivity and either having had mumps as a child or the median age at which mumps infection occurred (5.9 years amongst the *H. pylori* positive group, and 5.8 years amongst those who remained *H. pylori* negative).

## Discussion

In this study, we found that whilst adult *H. pylori* seropositivity was associated with socio-economic status and adverse housing conditions in early life, it was also associated with the timing of measles infection. However, similar associations were not seen for the other childhood infections studied.

Poverty, overcrowding and poor housing have been demonstrated to substantially increase the risk of communicable disease. However, while there were significant or near-significant associations between socio-economic status at birth and measles, mumps and chicken pox, these were not seen for socio-economic status later in childhood, or at all for housing deprivation score. An association was seen between housing conditions at age 5 years and socio-economic status at both birth and age 49-51 years and *H. pylori* seropositivity, with increasing odds with increasing deprivation. This is consistent with a number of studies that have found *H. pylori* to be associated with socio-economic disadvantage and poor and overcrowded housing in childhood [1-6].

We previously reported a protective effect of being breast fed on *H. pylori* risk [18], a finding confirmed by a later meta-analysis [19]. The lack of a socio-economic association in breast-feeding was reflective of the common use of breast feeding regardless of social status at the time the members of this cohort were born. This is also likely to reflect the conditions in middle and low-income countries where the protective effect of breast feeding was stronger in the meta-analysis [19]. Socio-economic status is a marker of general deprivation, including housing quality and hygiene and demonstrates a need for lessening deprivation, alongside increased use of breast-feeding, to reduce the short and long-term impacts of *H. pylori* infection.

Measles, mumps and chicken pox, are not predominantly spread faecal-oral or oral-oral routes, they are similar to *H. pylori* in that close association with an infected child is required for their acquisition. There was an association between adult *H. pylori* seropositivity and early measles infection in pre-school subjects, which did not persist for measles or other infections in later childhood. These data support the hypothesis that *H. pylori* colonization in British adults was acquired in early childhood and raises the possibility that viral infections such as measles may predispose to the development of *H. pylori* colonization. The specific interaction between measles and *H. pylori* seropositivity raises the possibility of a specific viral effect on the gastrointestinal mucosa or a more general interaction with the immune system. Measles has been described as causing a suppression in cell mediated immunity (although this is contested [20,21]) and potentially this may aid the colonization process with *H. pylori*[22]. These hypotheses require immunological and virological confirmation which is not possible with our cohort. The specific infections included in this study, measles, chickenpox and mumps, were those in which we were confident that a clinical diagnosis would have been appropriately made and recorded, and in which the lack of a positive diagnosis indicated non-infection. The lack of serological evidence of infection to support the clinical diagnosis of measles, chicken pox and mumps, remains a weakness of the study. However, historically, serological confirmation of clinical diagnoses would not have been considered.

Of the four infectious diseases that we have studied, it appears that *H. pylori* differs from the others by the strength of association with socio economic deprivation in early childhood.

## Conclusions

The original aim of the Newcastle Thousand Families study was primarily to demonstrate the associations between deprivation and morbidity and mortality within childhood [14]. Our findings further highlight the complex interaction between measles, childhood infections and other non-microbiological factors that occur within a whole population [23,24,6]. These data suggest a strong association between *H. pylori* and deprivation and raise the possibility of an interaction between early measles exposure and increased risk of *H. pylori* infection.

## Abbreviations

95% CI: 95% Confidence interval; H. pylori: Helicobacter pylori; OR: Odds ratio.

## Competing interests

The authors declare that they have no competing interests.

## Authors’ contributions

MSP conceived the idea for this analysis. LP led the data collection at age 49-51 years. DIC and JET were responsible for the *H. pylori* measurements. KDM and MSP analysed the data. MSP drafted the manuscript with contributions from all other authors. All authors approve the submission of this manuscript.

## Pre-publication history

The pre-publication history for this paper can be accessed here:

http://www.biomedcentral.com/1471-2334/13/422/prepub
